# Acceptability of Human Papilloma Virus Self-Sampling for Cervical Cancer Screening in a Cohort of Patients from Romania (Stage 2)

**DOI:** 10.3390/jcm11092503

**Published:** 2022-04-29

**Authors:** Mihaela Grigore, Ingrid-Andrada Vasilache, Petru Cianga, Daniela Constantinescu, Odetta Duma, Roxana Daniela Matasariu, Ioana-Sadiye Scripcariu

**Affiliations:** 1 Department of Obstetrics and Gynecology, ‘Grigore T. Popa’ University of Medicine and Pharmacy, 700115 Iasi, Romania; mihaela.grigore@umfiasi.ro (M.G.); roxana.matasariu@umfiasi.ro (R.D.M.); isscripcariu@gmail.com (I.-S.S.); 2 Department of Immunology, ‘Grigore T. Popa’ University of Medicine and Pharmacy, 700115 Iasi, Romania; petru.cianga@umfiasi.ro (P.C.); daniela.constantinescu@umfiasi.ro (D.C.); 3 Department of Epidemiology, ‘Grigore T. Popa’ University of Medicine and Pharmacy, 700115 Iasi, Romania; odetta.duma@umfiasi.ro

**Keywords:** self-sampling, cervical cancer screening, acceptability, HPV detection

## Abstract

(1) Background: Low patient’s adherence to conventional cervical cancer screening methods determined the need to take into consideration alternative approaches, and vaginal HPV self-sampling is one of them. We aimed to evaluate, using an online survey, the Romanian women’s acceptability of vaginal HPV self-sampling. (2) Methods: A 13-questions online survey was distributed on three Facebook groups, and the results were summarized. (3) Results: Despite of good educational background, 10.8% (*n* = 60) of the respondents did not know what a Pap smear is, and 33% (*n* = 183) were not informed about the free national cervical cancer screening program. Multivariate analysis revealed an increased likelihood of vaginal self-sampling acceptance among respondents who did not know about Pap test (OR: 7.80; 95%CI: 1.062–57.431; *p* = 0.021), national cervical cancer screening program (OR: 1.96; 95%CI: 1.010–3.806; *p* = 0.02), HPV infection (OR: 7.35; 95%CI: 3.099–17.449; *p*< 0.001) or HPV test (OR: 1.67; 95%CI: 0.950–2.948; *p* = 0.03). Moreover, women who did not previously undergo a cervical cancer screening program were more likely to accept the new screening method (OR: 1.62; 95%CI: 0.878–3.015; *p* = 0.04). (4) Conclusions: Our results showed high acceptability rates of vaginal HPV self-sampling among participants.

## 1. Introduction

With an estimated 604,000 new cases and 342,000 deaths worldwide in 2020, cervical cancer is the fourth most commonly diagnosed cancer and the fourth major cause of cancer death in women [1]. Each year in Romania approximately 1800 women die from cervical cancer, and almost 3400 receive this diagnosis [2,3]. The high incidence and mortality rates associated with cervical cancer place this country in the top of the European Union statistics [4].

Human papillomavirus (HPV) infection, some sexually transmittable infections (HIV and Chlamydia trachomatis), smoking, high parity, and long-term use of oral contraceptives are among the risk factors for developing cervical cancer [5,6]. HPV oncogenic types 16 and 18, responsible for almost 70% of the cervical neoplasia cases [7], are the main target of public health strategies for eradicating cervical cancer.

Because of the highly efficient primary (HPV vaccine) and secondary (screening) prevention strategies, cervical cancer is regarded as a preventable disease. However, low patients’ adherence to the national screening programs, mainly due to the lack of information and understanding, represented a barrier for achieving consistent results [8].

In recent years, vaginal HPV self-sampling emerged as a promising alternative to the conventional screening strategies based on Pap smears and/or HPV DNA testing. HPV self-sampling is a screening procedure that involves using a kit to collect cervico-vaginal samples, which are then sent to a laboratory for genotyping high-risk oncogenic HPV strains. It is easy to use by patients at home, is cost-effective, and has good overall accuracy in detecting HPV strains. Although recent studies have indicated that provider-collected cervical samples had the highest HPV-DNA sensitivity (84–100%), self-collected vaginal HPV-DNA tests appeared to have good performance, with a sensitivity ranging from 66 to 88% [9,10,11]. If the patient receives a positive result for the HPV test, she will establish an appointment with the gynecologist for deciding the best therapeutic plan.

Furthermore, self-sampling was found to be superior to HPV DNA testing performed by a clinician in terms of acceptance and preference [12,13]. The main benefits cited for vaginal self-sampling were less pain or physical discomfort, convenience, ability to perform the test in private, and less embarrassment or anxiety [14,15,16,17,18].

The challenges associated with the implementation of vaginal self-sampling include: difficulties regarding explaining the self-sampling procedure to participating women, specimen transportation and laboratory processing, as well as follow-up of positive women [19]. The provision of clear instructions accompanied by illustrations in suitable language would be a feasible approach for facilitating the self-sampling procedure [20,21].

On the other hand, the SARS-CoV-2 pandemic has accelerated the introduction of HPV self-sampling as a measure to increase the addressability to cervical cancer screening program, and self-sampling is included in the World Health Organization’s (WHO) recently issued guidelines on self-intervention for health, as well as the cervical cancer screening guidelines [22]. A recent study assessed the effectiveness of HPV self-sampling for cervical cancer screening during the SARS-CoV-2 pandemic, and found a high concordance for HPV detection between self-sampled and clinician-sampled specimens (90.2%), as well as a high willingness to repeat the procedure under the same conditions (89.2% of the participants) [23].

The main determinant of this study was the lack of literature data on the acceptability of doing self-sampling in the local population. We aimed to evaluate, using an online survey, the Romanian women’s acceptability of vaginal HPV self-sampling.

## 2. Materials and Methods

Between 15 February and 16 March 2022, data were collected using a Qualtrics form prepared by the investigator and titled: ‘A survey for analyzing the Romanian women’s thoughts regarding vaginal HPV self-sampling’, which was posted on three Facebook groups (Mothers at first pregnancy, About kids, and Mothers from Iasi).

The survey consisted of 13 questions that addressed maternal characteristics (age, living environment, and level of education), cervical cancer screening topic (6 closed questions that evaluated the women’s knowledge and experience with cervical cancer screening), as well as the women’s acceptability of vaginal HPV self-sampling. The participants were also shown an illustration of a vaginal self-sampling device along with its instructions for use so that they could complete the questionnaire fully informed.

The questionnaire was posted once group administrators approved it, and participants were notified that their anonymous responses would be published in this study. Before beginning the survey, all participants were instructed that it was completely voluntary and anonymous, and that they could skip any questions they did not feel comfortable answering or exit at any time. Each participant was instructed to read and declare if she agreed or disagreed with the survey’s questions. No names or medical identifying numbers were recorded to safeguard the participants’ privacy. All procedures were followed in compliance with the applicable norms and legislation. The Institutional Ethics Committee of the University of Medicine and Pharmacy ‘Grigore T. Popa’ gave its approval to this investigation (No. 96/23.06.2021).

Data collected were translated from the Romanian language into standard English. Incomplete surveys were excluded from the study. The total number of questionnaires distributed allowed statistical analysis with a cut-off for the absolute error and the type one error of 5%.

Acceptability of vaginal self-sampling was defined as the target variable. Categorical parameters were expressed as numbers and percentages, and statistical comparisons were made using the Chi-square test. We used a multivariate logistic regressions analysis to explore the associations between demographic characteristics, knowledge about the cervical cancer screening, previous screening attendance, and the target variable. The statistical analysis was performed using SPSS software (version 28.0.1, IBM Corporation, Armonk, NY, USA). A *p* value of less than 0.05 was considered statistically significant.

## 3. Results

Five hundred and eighty-two questionnaires were obtained and analyzed. Only 556 were included in the study due to incomplete data. Table 1 and Table 2 describe the acceptability of vaginal self-sampling for cervical screening among our respondents.

The majority of the respondents belonged to the 30–40 years age group (*n* = 339; 60.9%%), and only a minority of respondents had more than 51 years (*n* = 28; 5%). Most participants lived in urban areas (*n* = 324; 58.2%), and had a bachelor degree (*n* = 264; 47.4%).

Despite of good educational background, 10.9% (*n* = 61) of the respondents did not know what a Pap smear was, and 33% (*n* = 184) were not informed about the free national cervical cancer screening program. The results from the univariate analysis showed a significant association between age, medium, level of education, and the acceptability of vaginal HPV self-sampling (*p* < 0.001).

The odds ratios (OR) and 95% confidence intervals (CI) from the multivariate logistic analysis are shown in Table 3. The women from the 20–30 years age group were the most likely to accept the new method of cervical cancer screening (OR: 15.78; 95% CI: 2.161–115.304; *p* = 0.003). There was also a good likelihood for accepting this screening method for women included in the 40–50 years age group (OR: 3.34; 95% CI: 1.020–10.960; *p* = 0.02).

Women living in rural areas (OR: 2.87; 95% CI: 1.483–5.563; *p* < 0.001), as well as those who possessed a high-school diploma (OR: 2.73; 95% CI: 1.478–5.057; *p* < 0.001), were more likely to accept the screening method. On the other hand, it appeared that respondents from urban areas (OR: 0.34; 95% CI: 0.180–0.674; *p* < 0.001), as well as those with a bachelor degree (OR: 0.28; 95% CI: 0.155–0.532; *p* < 0.001), were less likely to embrace the method.

An alarming number of respondents did not know what an HPV infection (*n* = 238; 42.8%) or HPV test (*n* = 267; 48%) was. Moreover, more than one third of the participants (*n* = 198; 35.6%) did not do a Pap test or an HPV test for cervical cancer screening during their lifetime. On the other hand, more than half of the women (*n* = 358; 64.3%) underwent cervical screening, and Pap smear was the most used screening method (*n* = 291; 81.2%).

Univariate analysis indicated a significant association between knowledge about Pap test (*p* = 0.006), national cervical screening program (*p* = 0.043), HPV infection (*p* < 0.001), previous participation to the screening program (*p* < 0.001) and the acceptability of vaginal HPV self-sampling.

Multivariate analysis revealed an increased likelihood of vaginal self-sampling acceptance among respondents who did not know about Pap test (OR: 7.80; 95%CI: 1.062–57.431; *p* = 0.021), national cervical cancer screening program (OR: 1.96; 95%CI: 1.010–3.806; *p* = 0.02), HPV infection (OR: 7.35; 95%CI: 3.099–17.449; *p* < 0.001) or HPV test (OR: 1.67; 95%CI: 0.950–2.948; *p* = 0.03). Moreover, women who did not previously undergo a cervical cancer screening program were more likely to accept the new screening method (OR: 1.62; 95%CI: 0.878–3.015; *p* = 0.04).

The majority of the respondents (*n* = 523; 94%) considered self-sampling a good alternative to Pap test for women who do not consult their physicians. However, 87% (*n* = 484) of the subjects agreed that cervical sampling performed by a doctor is better than vaginal self-sampling (*p* = 0.02).

64.7% (*n* = 360) of the participants agreed with the statement that most women will choose self-sampling over a visit to a doctor, while 81.6% of them considered HPV vaginal self-sampling a good alternative to the conventional screening method. The answers to the last two questions indicated the women’s preference of self-sampling over the doctors’ appointments, with a significant level of acceptability among the participants (*p* < 0.001).

## 4. Discussion

This observational retrospective study, based on a self-administered online survey, showed high acceptability rates of vaginal HPV self-sampling among participants, including under-screened women. Moreover, our study also confirmed the women’s lack of knowledge about cervical cancer screening, even though the majority of the respondents had higher education.

In terms of acceptability, our multivariate analysis revealed that the women from the 20–30 years age group, followed by those in the 40–50 years age group, were most likely to accept the new method of cervical cancer screening, while those women in the 30–40 years age group were significantly less likely to embrace the method (OR: 0.07; 95%CI: 0.023–0.241; *p* < 0.001). These findings could be explained by the fact that very young women are more open to new technologies, while those middle-aged, who are less used to regular gynecological appointments, are in favor of self-sampling in the comfort of their own home. Similar results were reported in various studies that outlined increased acceptability of vaginal self-sampling among younger generations of women due to decreased embarrassment, time/effort investment and a ‘do-it- yourself’ attitude, as well as older women, due to previous bad gynecological experiences or the need for privacy [10,24,25,26].

Our results also indicated that women living in rural areas, as well as those who possessed a high-school diploma, were more likely to accept the screening method, while those from urban areas and with a bachelor degree were less likely to accept it. Several studies demonstrated that women living in rural areas do not benefit as much as women from urban areas in terms of cervical cancer screening [27,28]. Limited accessibility to Pap smear and/or HPV testing, rural practice configuration or scarce financial resources were cited as factors that negatively influence the women’s addressability to cervical cancer screening programs [29,30]. On the other hand, developed sanitary infrastructure along with higher consumption of healthcare services in the urban areas could be considered reasons for the women’s preference to traditional cervical cancer screening [31,32]. Although the literature data are conflicting regarding the influence of the educational background over the HPV self-sampling acceptability [33,34,35], we hypothesize that women with at least a bachelor degree tend to have a higher compliance to conventional cervical cancer screening.

In Romania, there is an active national cervical cancer screening program developed by the Public Health Ministry in partnership with regional hospitals that offers free Pap testing for women aged between 25 and 64 years [36]. The lack of knowledge among the participants about the cervical cancer screening in Romania was a key finding of the study. An alarming number of respondents did not know what a Pap smear (*n* = 60; 10.8%), HPV infection (*n* = 237; 42.7%) or HPV test (*n* = 267; 48.1%) was despite a good educational background. These data are comparable to that outlined by various studies in developing or underdeveloped countries [37,38,39,40].

Our multivariate analysis revealed an increased likelihood of vaginal self-sampling acceptance among respondents who did not know about Pap tests, the national cervical cancer screening program, or HPV infection. Moreover, women who did not previously undergo a cervical cancer screening program appeared more likely to accept the new screening method. Our results are in line with those reported in an observational study by Lancrajan et al., which outlined the limited knowledge of Romanian women about cervical cancer screening [41]. Furthermore, previous studies indicated that self-sampling acceptability was higher among women who had never undergone cervical screening before, in both rich and underprivileged societies [42,43].

We hypothesize that women with scarce information regarding this topic would feel more comfortable with a self-administered test that does not require a visit to the gynecologist. This emphasizes the importance of a thorough education about the feasibility, benefits, and accuracy of self-sampling in order to increase screening participation.

In this study, vaginal self-sampling was considered a good alternative to Pap test for women who do not consult their physicians, with an acceptance rate of 89.7%. The main reasons for low addressability to the cervical cancer screening program in Romania were explored by Todor et al., and included the lack of national coverage and the penetration in the rural areas, mass-media promotion campaigns, funding, involvement of general practitioners, and bureaucracy, as well as program monitoring [44]. Our results showed that 33% (*n* = 183) of the respondents were not informed about the free national cervical cancer screening program. The present study confirmed the low adherence of Romanian respondents to the cervical cancer screening program, despite the high acceptability rates of HPV self-sampling among respondents.

Although the majority of respondents considered that cervical sampling performed by a doctor is better than vaginal self-sampling (86.9%), they have also expressed their preference to self-sampling over the doctors’ appointments (81.5% vs. 15.6%). Analysis of the patient’s preferences over these methods was not the purpose of this study, but the comfort, privacy, ergonomics, and accessibility of self-sampling were cited as some of the main reasons for choosing this alternative over a physician’s appointment [42,45,46,47].

A recent systematic review by Devarapalli et al., which evaluated the most important barriers associated with cervical cancer screening in low and middle-income countries, concluded that the following elements are important obstacles to fulfilling the screening’s objectives: psychological, structural, sociocultural, and religious barriers, as well as lack of knowledge and awareness [48]. All these aspects must be considered when establishing public health programs in order to improve the campaigns’ outcomes.

Several studies have shown that women’s awareness of cervical cancer, HPV, and its vaccine can effectively improve the mortality rates and reduce incidences of this disease [49,50,51]. Therefore, effective measures to improve women’s level of knowledge should include all new means of information (mass-media, social media platforms, news websites, etc.) that allow dissemination of relevant data on a regular basis. Moreover, telemedicine has been shown to be a useful tool for providing medical advice during the SARS-CoV-2 pandemic, and could be further added to the screening programs [52,53].

The participants’ profile, which may not be representative of under-screened women targeted by vaginal self-sampling, is one main weakness in our study. The majority of the subjects had a high educational level and a high rate of cervical cancer screening compliance. This could be explained by the way women who had access to social media platforms were recruited. We were not able to generalize the findings of this study since our participants were limited to respondents attending social media groups. Other limitations of our study include: small number of participants included, short time-frame for data collection, and restricted information concerning the epidemiological characteristics of the respondents. We chose to design this survey with only a limited number of questions that allowed a reasonable completion rate, and a 3 min response time. Similar studies, that used web-based surveys disseminated through Facebook groups, acknowledged the difficulties regarding the maintenance of an active interest for the survey and representativeness of epidemiological data [54,55].

Further research is needed to evaluate the acceptability of vaginal HPV self-sampling in various cohorts of patients, especially those who manifest low compliance to conventional screening methods.

## 5. Conclusions

This study demonstrated high acceptability rates of vaginal HPV self-sampling among respondents, indicating this as a feasible method for increasing the women’s compliance to cervical cancer screening.

However, an alarming number of women did not have knowledge about cervical cancer and its screening possibilities. Therefore, more education and public health interventions are needed to raise awareness about this topic.

Future studies could further evaluate the values and preferences of Romanian women regarding the secondary prevention of cervical cancer.

## Figures and Tables

**Table 1:** The acceptability of vaginal HPV self-sampling among the respondents, considering the demographic characteristics and cervical cancer screening knowledge.

Variable		Self-Sampling Acceptance (*n*/%)		Total Number of Responses (n/%)	*p* Value
		Yes	No		
Age	20–30 years	108 (19.4%)	1 (0.1%)	109 (19.6%)	<0.001
	30–40 years	285 (51.2%)	54 (9.7%)	339 (69.9%)	
	40–50 years	78 (14%)	3 (0.5%)	81(14.5%)	
	>51 years	27 (4.8%)	1 (0.1%)	28 (5%)	
Medium	Urban	282 (50.7%)	42 (7.5%)	324 (58.3%)	<0.001
	Rural	216 (38.8%)	16 (2.8%)	232 (41.7%)	
Level of education	Primary school (≤4 years of study)	3 (0.5%)	1 (0.1%)	4 (0.7%)	<0.001
	Pre-high school (5–8 years of study)	26 (4.6%)	1 (0.1%)	27 (4.8%)	
	High-school (9–12 years of study)	246 (44.2%)	15 (2.6%)	261 (46.9%)	
	≥Bachelor degree	222 (39.9%)	42 (7.5%)	264 (47.4%)	
Do you know what Pap test is?	Yes	438 (78.7%)	57 (10.2%)	495 (89%)	0.006
	No	60 (10.7%)	1 (0.1%)	62 (11%)	
Do you know that in Romania there is a free national program for Pap testing?	Yes	327 (58.8%)	45 (8.1%)	372 (66.9%)	0.043
	No	171 (30.8%)	13 (2.3%)	184 (33.1%)	
Do you know what Human papilloma virus (HPV) infection is?	Yes	267 (48%)	51 (9.1%)	318 (57.1%)	<0.001
	No	231 (41.5%)	7 (1.2%)	238 (42.9%)	
Do you know what HPV test is?	Yes	252 (45.3%)	37 (6.6%)	289 (51.9%)	0.072
	No	246 (44.2%)	21 (3.7%)	267 (48.1%)	
Did you previously do a Pap test or HPV test for cervical cancer screening?	Yes	315 (56.6%)	43 (7.7%)	358 (64.3%)	<0.001
	No	183 (32.1%)	15 (2.6%)	198 (35.7%)	
If your previous answer was yes, which test did you take?	Pap test	264 (47.4%)	27 (4.8%)	291 (81.2%)	<0.001
	HPV test	3 (0.5%)	1 (0.1%)	4 (1.1%)	
	Both	45 (8%)	18 (3.2%)	63 (17.7%)	

**Table 2:** The acceptability of vaginal HPV self-sampling among the respondents, considering their opinions regarding cervical cancer screening.

Variable		Self-Sampling Acceptance (*n*/%)		Total Number of Responses (n/%)	*p* Value
		Yes	No		
Do you consider self-sampling a good alternative to Pap test for women who do not consult their physicians?	Yes	498 (89.5%)	25 (4.4%)	523 (94.1%)	<0.001
	No	0 (0%)	30 (5.4%)	30 (5.4%)	
	I have no opinion	0 (0%)	3 (0.5%)	3 (0.5%)	
I consider that cervical sampling performed by a doctor is better than self-sampling	Yes	430 (77.3%)	54 (9.7%)	484 (87%)	0.02
	No	39 (7%)	0 (0%)	39 (7%)	
	I have no opinion	30 (5.4%)	3 (0.5%)	33 (6%)	
I consider that most women will choose self-sampling over a visit to a doctor	Yes	339 (61%)	21 (3.7%)	360 (64.7%)	<0.001
	No	109 (19.6%)	27 (4.8%)	136 (24.4%)	
	I have no opinion	51 (9.1%)	9 (1.6%)	60 (10.7%)	
I consider self-sampling a good alternative and I would use it instead of going to the doctor	Yes	442 (79.4%)	12 (2.1%)	454 (81.6%)	<0.001
	No	45 (8.1%)	42 (7.5%)	87 (15.6%)	
	I have no opinion	12 (2.1%)	3 (0.5%)	15 (21.7%)	

**Table 3:** Multivariate logistic regression analysis to identify factors associated with the acceptability of HPV self-sampling.

Variable		Self-Sampling Acceptance			*p* Value
		Odds ratio	Lower Bound CI	Upper Bound CI	
Age	20–30 years	15.78	2.161	115.304	0.003
	30–40 years	0.07	0.023	0.241	<0.001
	40–50 years	3.34	1.020	10.960	0.02
	>51 years	3.26	0.436	24.503	0.12
Medium	Urban	0.34	0.180	0.674	<0.001
	Rural	2.87	1.483	5.563	<0.001
Level of education	Primary school (≤4 years of study)	0.34	0.035	3.376	0.18
	Pre-high school (5–8 years of study)	3.26	0.436	24.503	0.12
	High-school (9–12 years of study)	2.73	1.478	5.057	<0.001
	≥Bachelor degree	0.28	0.155	0.532	<0.001
Did women know about Pap test?	Yes	0.12	0.017	0.942	0.021
	No	7.80	1.062	57.431	0.021
Did women know about the free national screening program?	Yes	0.50	0.263	0.990	0.02
	No	1.96	1.010	3.806	0.02
Did women know about Human papilloma virus (HPV) infection?	Yes	0.13	0.057	0.323	<0.001
	No	7.35	3.099	17.449	<0.001
Did women know about HPV test?	Yes	0.59	0.339	1.053	0.03
	No	1.67	0.950	2.948	0.03
Were women previously screened for cervical cancer?	Yes	0.61	0.332	1.139	0.04
	No	1.62	0.878	3.015	0.04

## Data Availability

The data presented in this study are available on request from the corresponding author. The data are not publicly available due to local policies.
